# Segmentation of Cemento-Osseous Dysplasias Using an Artificial Intelligence Algorithm

**DOI:** 10.1016/j.identj.2025.109340

**Published:** 2026-01-03

**Authors:** Duygu Çelik Özen, Oğuzhan Altun, Şuayip Burak Duman, İbrahim Şevki Bayrakdar

**Affiliations:** a Department of Oral and Dentomaxillofacial Radiology, Faculty of Dentistry, Inonu University, Malatya, Turkey; b Department of Diagnostic Sciences, Texas A&M College of Dentistry, Dallas, Texas, USA; c Department of Oral and Dentomaxillofacial Radiology, Faculty of Dentistry, Eskisehir Osmangazi University, Eskisehir, Turkey

**Keywords:** Artificial Intelligence, Cemento-osseous dysplasia, Cone beam computed tomography, Deep learning

## Abstract

**Introduction and Aims:**

In recent years, artificial intelligence (AI) has emerged as a powerful tool in medical imaging and in the analysis of complex bone pathologies such as cemento-osseous dysplasias. The aim of this study is to perform segmentation of cemento-osseous lesions using AI algorithms on cone beam computed tomography (CBCT) images and to evaluate the diagnostic performance of a diagnostic AI model designed for the diagnosis of cemento-osseous dysplasias.

**Methods:**

In this study, cone beam computed tomography (CBCT) images taken for various reasons in radiology archive Department of Oral and Maxillofacial Radiology were retrospectively reviewed. As a result of radiographic evaluation, images recorded in the archive with at diagnosis of cemento-osseous dysplasias were determined. Fifty DICOM images were uploaded to the 3D slicer software, and cemento-osseous dysplasias were polygonally labeled and saved in Neuroimaging Informatics Technology Initiative (NIfTI) format. The nnU-Net v2-based automated algorithm for lesion segmentation was developed using the CranioCatch (CranioCatch, Eskişehir) software program using the PyTorch library in the Python framework (v3.6.1; Python Software Foundation). 80% of the data was used for training, 10% for validation and 10% for testing. The results were evaluated according to the criteria of precision, sensitivity, Dice Coefficient, Jaccard Index.

**Results:**

The precision, sensitivity, Dice Coefficient and Jaccard Index for the segmentation of cemento-osseous dysplasias were 0.805, 0.889, 0.839, and 0.730, respectively.

**Conclusions:**

The model we used achieved successful results in cemento-osseous dysplasias segments. The results of this planned study are promising in terms of providing a guidance for physicians in diagnosis.

**Clinical Relevance:**

Automated segmentation of cemento-osseous lesions, where radiological images play a critical role in both diagnosis and follow-up, has the potential to enable precise and consistent definition of lesion boundaries and standardize the follow-up process, enabling more reliable data for long-term studies.

## Introduction

Cemento-osseous dysplasias are the most commonly observed benign fibro-osseous lesions of the jawbones. Generally observed in the periapical regions of teeth, they can also be found in edentulous alveolar processes.^1^^,^^2^ There are 3 well-known subtypes: periapical, focal, and florid and according to the 2022 classification by the World Health Organization, familial florid cemento-osseous dysplasia, which is extremely rare and inherited in an autosomal dominant manner, has been included as the fourth subtype in this group.^3^ Although these lesions have many common features radiographically and microscopically, they are differentiated from each other based on some clinical features and location in the jaws.^4^ Most lesions usually do not cause any symptoms unless they are infected and are seen incidentally during routine radiographic examination. Cemento-osseous dysplasias usually do not require treatment; however, radiologic and clinical follow-up examinations are important.^4^

Artificial intelligence (AI)-based automated systems, which are frequently used today, have made the analysis of radiological images faster, more accurate, and more efficient. The use of AI in dentistry first started with the integration of digital imaging techniques and databases, and has expanded over time with the use of machine learning algorithms in areas such as diagnosis, decision making, treatment planning and prediction of treatment outcomes.^5^ Machine learning is a subfield of AI that aims to develop computer models capable of learning from presented data and making independent predictions. Machine learning-based models increase their accuracy by continuously improving their performance through the data obtained.^6^ Recently, deep learning has made tremendous progress, reportedly outperforming humans in object recognition and visual tasks.^7^ Convolutional neural networks (CNN) are the most popular deep learning approach based on the visual cortex of living things, and many architectures used today are built on CNN.^8^ In recent years, AI has gained increasing importance in dentistry by enhancing diagnostic accuracy, supporting treatment planning, and facilitating clinical decision-making. According to Samaranayake et al.,^9^ AI has great potential to transform dental practice, particularly through its ability to interpret radiographic images, detect pathologies, and assist clinicians in planning effective treatments.

In the clinical practice of dentistry, 3-dimensional cone beam computed tomography (CBCT) images are often used to aid diagnosis, treatment planning and surgery. CBCT imaging provides comprehensive 3-dimensional volumetric information about teeth and alveolar bone.^10^ In the presence of multiple cemento-osseous dysplasias in the jaw bones, CBCT examination is needed to evaluate the internal structure of the lesions and their impact on adjacent anatomical structures in orthogonal sections. Recently, CBCT has been shown to be a valuable tool in the diagnosis and evaluation of cemento-osseous dysplasias.^2^^,^^11^^,^^12^ However, to the best of our knowledge there is no study in the literature in which semeto-osseous lesions are detected with AI models.

The aim of this study is to perform segmentation of cemento-osseous dysplasias, in which radiological images play an important role in diagnosis and follow-up, using an AI model on CBCT images.

## Methods

The protocol of this retrospective study was in accordance with all the principles of the Declaration of Helsinki and access to the data used was restricted to the principal investigators. The study was approved by the Inonu University Non-Interventional Clinical Research Ethics Committee decision dated October 31, 2023 and numbered 2023 to 5087. Before each CBCT scan, written informed consent was obtained from all participants included in the study.

### Patient selection

For this study, data from 2018 to 2024 in the archive of the Oral and Dentomaxillofacial Radiology Department of Inonu University Faculty of Dentistry were retrospectively reviewed by a doctor (DCO with 3 years of experience). All images in the archive were acquired with a field of view (FOV) of 15 × 12 cm^2^ with exposure parameters of 110 kVp, 1 to 20 mA, scan time of 18 s and voxel size of 0.2 mm. Images archived with a diagnosis of cemento-osseous dysplasia were identified based on radiologic, clinical, and, when available, histopathological data. Images with cemento-osseous dysplasias were excluded if they were in the initial (radiolucent) or sclerotic (radiopaque) phase of the lesion. Only images in the mixed (radiolucent/radioopaque) phase of the lesion were included in the study.

The study population consisted of 50 patients between the ages of 25 to 72 (mean age 46.3 years), 46 females and 4 males. In each patient, cemento-osseous dysplasias were classified based on their location and extent in the jawbone as follows:

Florid cemento-osseous dysplasia: Multifocal with bilateral mandibular involvement,

Focal cemento-osseous dysplasia: Unilateral involvement in the periapical region of the posterior mandible,

Periapical cemento-osseous dysplasia: Located in the periapical region of the anterior mandible.

Of the cemento dysplasias, 2 were periapical cemento-osseous dysplasia, 22 were focal cemento-osseous dysplasias and 26 were florid cemento-osseous dysplasias. One of the focal cemento-osseous dysplasia was localized in the maxilla, and 2 of the floride cemento-osseous dysplasias were localized in both the maxilla and mandible; all other lesions were localized in the mandible.

Participants over 18 years of age who presented with at least one cemento-osseous dysplasias located in the maxilla or mandible and exhibiting a mixed radiographic stage were included in the study. Images were excluded if they were nondiagnostic due to inadequate quality caused by metal artifacts, improper patient positioning, or motion artifacts during image acquisition. Furthermore, images demonstrating fracture lines, the presence of foreign objects, lesions other than cemento-osseous dysplasias, or radiographic features suggestive of craniofacial syndromes were excluded from the analysis.

### Evaluation and labeling of images

CBCT images of 50 patients were anonymized and saved as Digital Imaging and Communication in Medicine (DICOM) files. The evaluation of DICOM images was performed 2 Oral and Maxillofacial Radiolog (DCO with 3 years of experience and SBD with 12 years of experience) at the CBCT reporting room of Inonu University Faculty of Dentistry, using a Precision 3640 Tower CTO BASE workstation (Intel(R) Xeon(R) W‐1250P (6 cores, 12M cache, base processor frequency 4.1 GHz, Max Turbo Frequency 4.8 GHz), DDR4‐2666, 64GB DDR4 (4 × 16GB) 2666 MHz UDIMM ECC Memory capacity, 256GB SSD SATA, Nvidia Quadro P620, 2GB) (Dell) and a 27," 1920 × 1080 pixel IPS LCD monitor (Dell).

To check how reliable the observers were, they each looked at 20 randomly chosen cases on their own and then looked at them again 4 weeks later. Cohen's κ coefficient (κ = 0.87) was used to measure how consistent the observers were with each other. According to the Landis and Koch scale, this means that they were almost perfectly in agreement.

DICOM files of 50 patients were transferred to the 3D Slicer imaging software for segmentation. 3D Slicer is a free and open source software package that is frequently used in areas such as labeling, image analysis and scientific visualization (www.slicer.org).

**Fig. 1:**
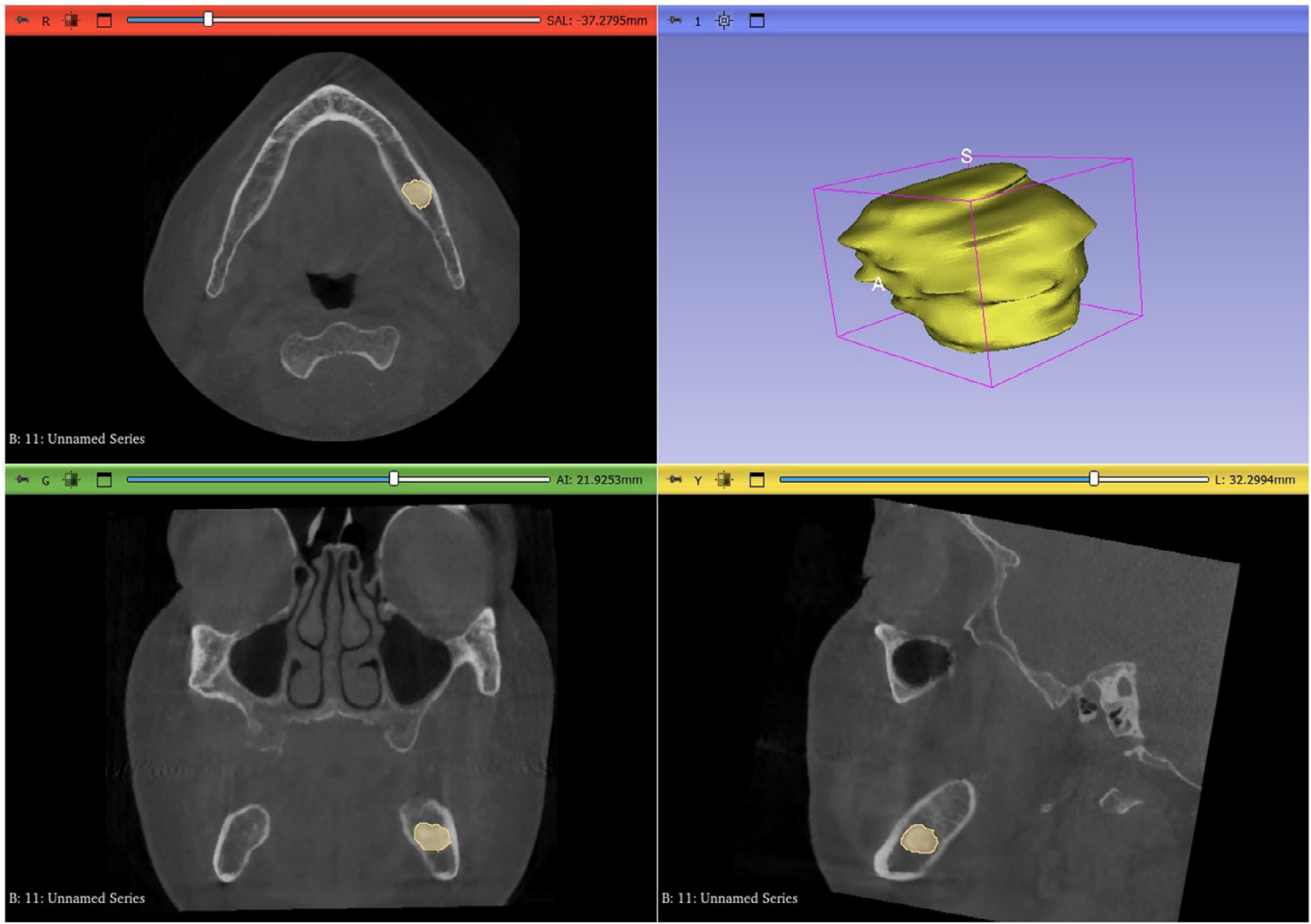
Labeling of cemento-ossous dysplasias on 3D slicer program.

Labeling is the process of identifying an area in an image and determining the region to which the object belongs. DICOM images were labeled in all 3 planes (axial, sagittal and horizontal) with a manual polygonal technique using the segment editor option in the 3D Slicer software program (Figure 1). In some lesion types where cemento-osseous dysplasias merge with tooth roots, the tooth roots are not included in the labeling area.

Upon completion of all labeling procedures, the labels were checked by 2 oral and maxillofacial radiologists (IBS and SBD with 12 years of experience) and full agreement was reached on all labels.

After labeling, the images were converted to the Neuroimaging Informatics Technology Initiative (NIfTI) format with the 'segmentation' option and saved.

### Development of deep learning model

nnU-Net v2 based automatic cemento-osseous dysplasia segmentation algorithm was developed using the PyTorch library in the Python framework (v3.6.1; Python Software Foundation) with the software program CranioCatch.The model was trained for 1000 epochs with a learning rate of 0.00001, using a 128 × 128 × 128 voxel patch size and a batch size of 2. During training, the Stochastic Gradient Descent (SGD) optimizer was used with an initial learning rate of 0.01, momentum of 0.99, Nesterov acceleration, and a weight decay of 3 × 10^−^⁵. The loss function, defined as a combination of Dice and cross-entropy losses via the DeepSupervisionWrapper, effectively mitigated class imbalance by assigning higher gradient contributions to underrepresented lesion voxels. Input CBCT images were normalized using nnU-Net’s standard CTNormalization method, and no masking was applied.

**Fig. 2:**
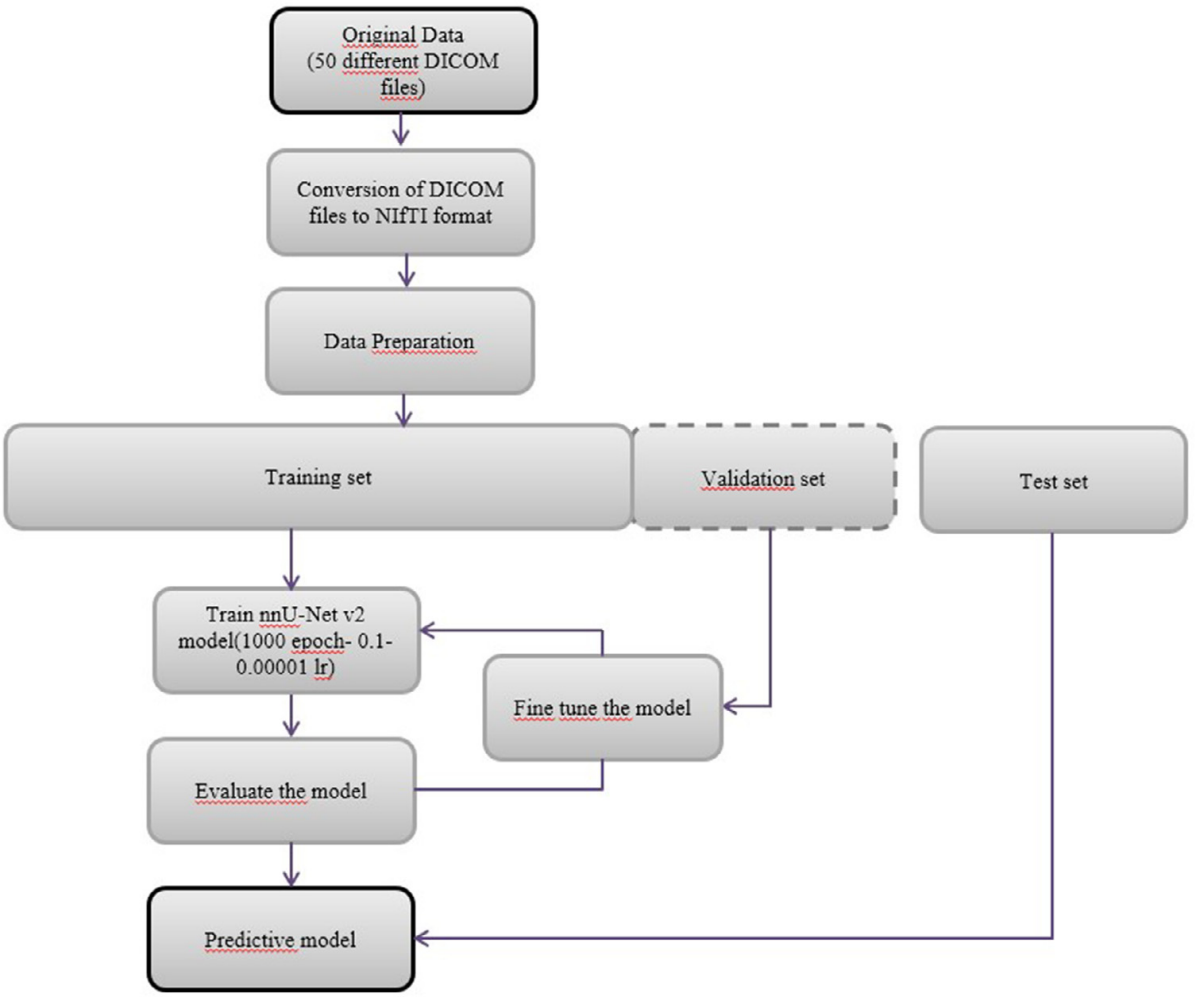
Diagram of the cemento-osseous dysplasias segmentation model performed with the nnU-Net v2 model.

**Fig. 3:**
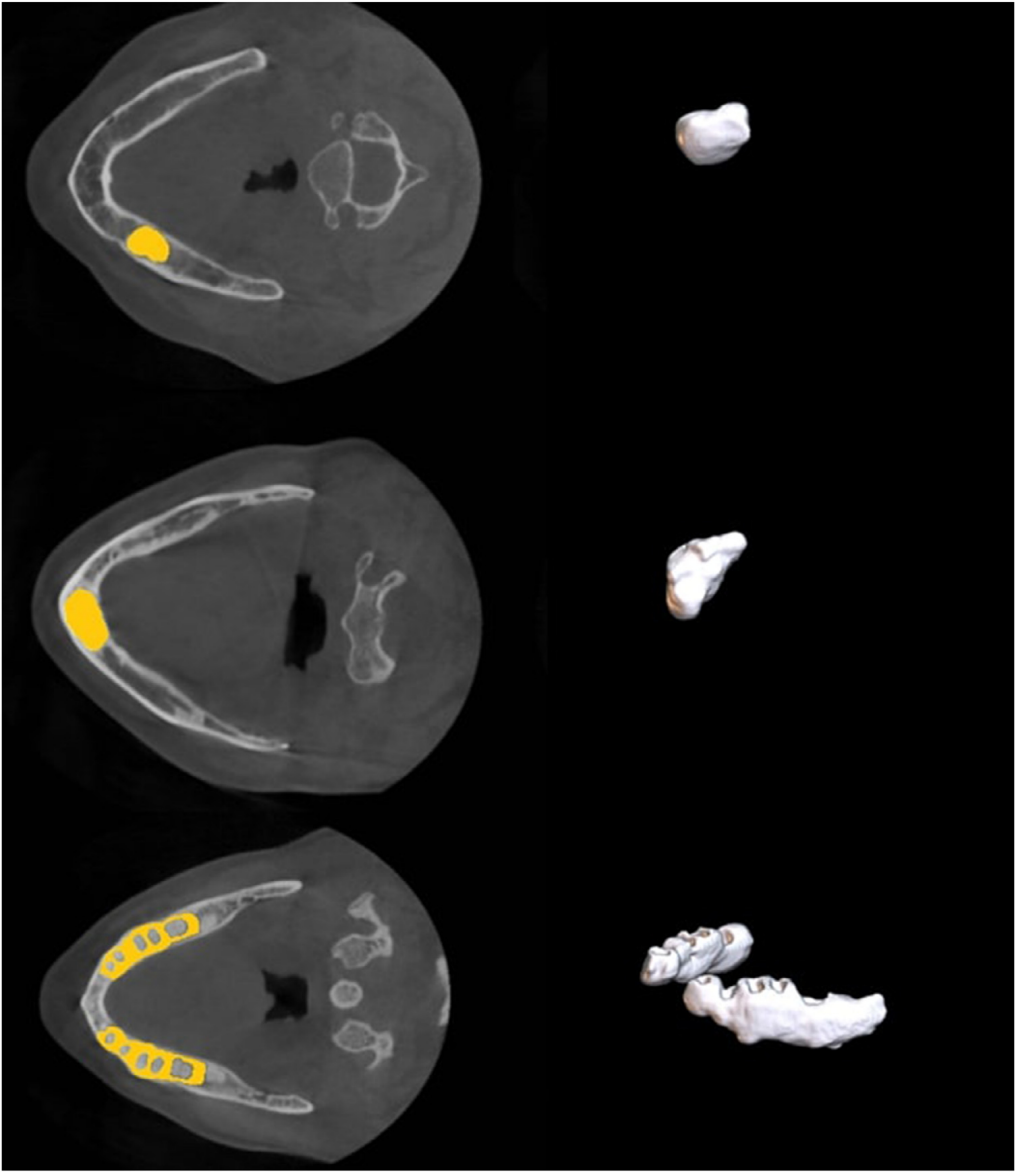
Three-dimensional lesion images to be used for model training obtained from labeled images of cemento-osseous dysplasias.

To further address class imbalance and improve model generalization, comprehensive data augmentation techniques were employed. Random rotations of up to ±30° were applied along each axis, and images were randomly scaled between 0.7 × and 1.4 ×, while elastic deformation was not used. Gaussian noise (10% probability, variance 0-0.1) and Gaussian blurring (20% probability, with varying sigma values per channel) were applied. Brightness and contrast were randomly adjusted (15% probability, multiplier range 0.75-1.25), and low-resolution simulation was performed by downsampling and upsampling samples with a 25% probability. Gamma correction was applied with 2 different probability–parameter settings, and all samples were randomly mirrored along the x, y, and z axes. These augmentations enabled the network to better learn rare lesion voxels under diverse spatial and intensity conditions. The encoder–decoder architecture consisted of 2 convolutional layers at each stage, with feature maps increasing from 32 at the base level to 320 at the highest level, and pooling operations applied at 6 levels with a 3 × 3 × 3 kernel size. Figure 2 shows a diagram of the segmentation model of the nnU- Netv2 cemento-osseous dysplasia and Figure 3 shows the 3-dimensional version obtained from the labeled image. The mathematical operations in the training of the model were performed at the Dental AI Laboratory of Eskisehir Osmangazi University Faculty of Dentistry with Dell PowerEdge T640 Computer Server (Dell Inc.), Dell PowerEdge T640 GPU Compute Server (Dell Inc.) and Dell PowerEdge R540 Storage Server (Dell Inc.).

### Segmentation with nnU-Net v2

**Fig. 4:**
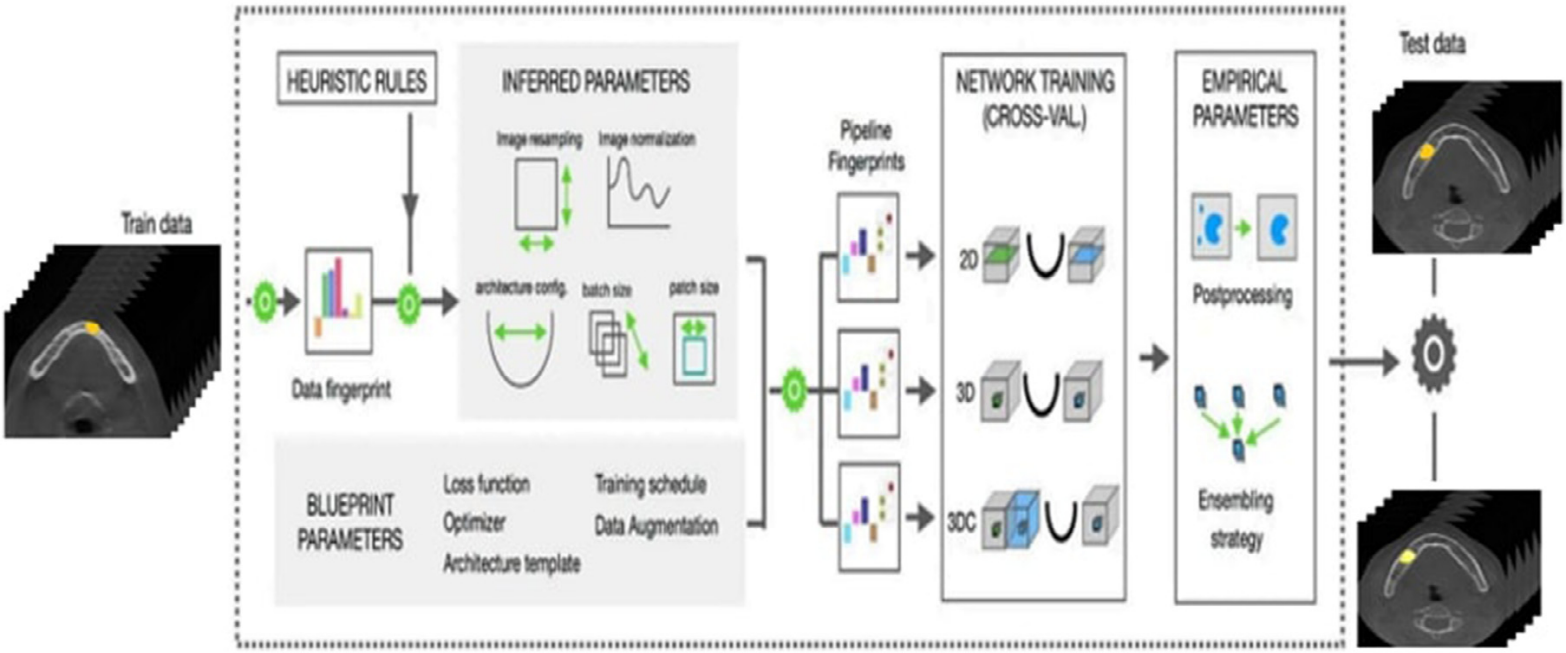
nnU-Net v2 architecture.

The nnU-Net v2 framework was selected because it can automatically modify training parameters, network architecture, and preprocessing steps based on dataset properties, resulting in consistent and repeatable segmentation results without requiring human optimization. Previous research by Isensee et al.^13^ has also confirmed its adaptability to CBCT segmentation.^14^ nnU-Net v2 uses heuristics to analyze training data and identify data-dependent hyperparameters, so-called “data fingerprints.” Pipeline fingerprints are created by combining plan parameters such as loss function, optimizer and architecture template, and inference parameters such as image resampling, image normalization, batch size and patch size with the data fingerprint. These pipeline fingerprints facilitate network training for 2D and 3D cascaded U-Net models by using predefined hyperparameters. By combining various network configurations with postprocessing techniques, the average “Dice coefficient” for training data is optimized. The most efficient structure is then used to generate predictions from the test data (Figure 4).^13^^,^^15^

The preprepared training set was input into the network, where forward propagation calculated the output of the model for each data set. Input images are paired with binary segmentation masks, where object pixels are labeled as 1s and background pixels as 0s, creating a black-and-white representation for precise object identification. The loss function quantifies the difference between the predicted segmentation and ground truth, and the gradients are computed via back-propagation to adjust the model parameters. The optimizer updates the weights and biases of the model to minimize losses. After each training epoch, the performance was evaluated on a validation set, and hyperparameters were adjusted accordingly. The training concludes when the validation performance plateaus. Finally, the test set is used to generate segmentation results, which are assessed using performance metrics.^16^ In this study, 80% of the dataset was randomly assigned to the training, 10% to the validation and 10% to the test dataset. The training and validation sets were used to generate and estimate the optimal CNN algorithm weight factors, and the test data set was used to evaluate the success of the models.

### Evaluation of model performance

True positive (TP): Number of correct detections. Situation in which the observer's prediction matches the model's prediction

False positive (FP): The model produce an incorrect result according to the observer's prediction

False negative (FN): The number of regions where the observer prediction was found but the model did not.

True negative (TN): The number of regions where there is no observer prediction and the algorithm does not find it.

The precision and sensitivity values were obtained from these metrics. The Jaccard index and Dice coefficient were calculated for the overlap between the ground truth and predicted segmentation maps, as well as the receiver operating characteristic curve (ROC) curve and area under curve (AUC).

#### Sensitivity (recall)

It measures the capacity of the algorithm to capture and correctly classify true positive examples. Higher sensitivity values indicated an increased ability of the algorithm to successfully detect positive examples within the dataset. The formula is as follows;

$$Sensitivity=\frac{\mathrm{TP}}{\mathrm{TP}+\mathrm{FN}}$$

#### Precision

It measures the proportion of samples predicted as positive that are actually positive. A higher precision value indicates that the model produces fewer false positive predictions. Its formula is as follows;

$$Precision=\frac{\mathrm{TP}}{\mathrm{TP}+\mathrm{FP}}$$

#### Dice coefficient

**Fig. 5:**
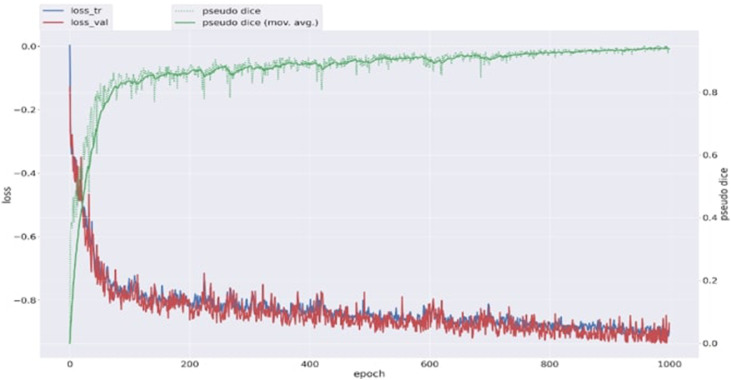
Dice coefficient evolution of the training and validation datasets during the training process. loss_tr: training set loss curve, loss_val: validation set loss curve, pseudo dice: validation set pseudo dice curve, pseudo dice (mov. avg.): moving average of validation set pseudo dice curve.

Following the formula where X represents a set representing the ground truth and Y represents the predicted segmentation set, the dice coefficient represents the relative overlap between X and Y. The intersection is the number of overlapping pixels between the predicted and actual segmentations. In the case of perfect agreement, the dice coefficient is 1, if there is no overlap the corresponding value is a dice coefficient of 0. Figure 5 shows a graph of the change in the dice coefficient values during model training. Its formula is as follows;

$$DiceCoefficient=\frac{2|X\cap Y|}{|X|+|Y|}$$

#### Jaccard index

The Jaccard Index is the area of overlap between the predicted segmentation and ground truth divided by the area of convergence between the predicted segmentation and ground truth. This metric ranges from 0 to 1, with 0 indicating no overlap and 1 indicating perfectly overlapping segmentation.

$$JaccardIndex=\frac{2|X\cap Y|}{|X\cup Y|}$$

#### ROC curve graph

The ROC curve was calculated as the proportion of true positives on the y-axis divided by the proportion of false positives on the x-axis. Because both the x and y axes have values between 0 and 1, it can take any value between 0 and 1. The closer the AUC is to 1, the better is the overall diagnostic performance of the test, and a test with an AUC of 1 is perfectly accurate.^17^

## Result

**Fig. 6:**
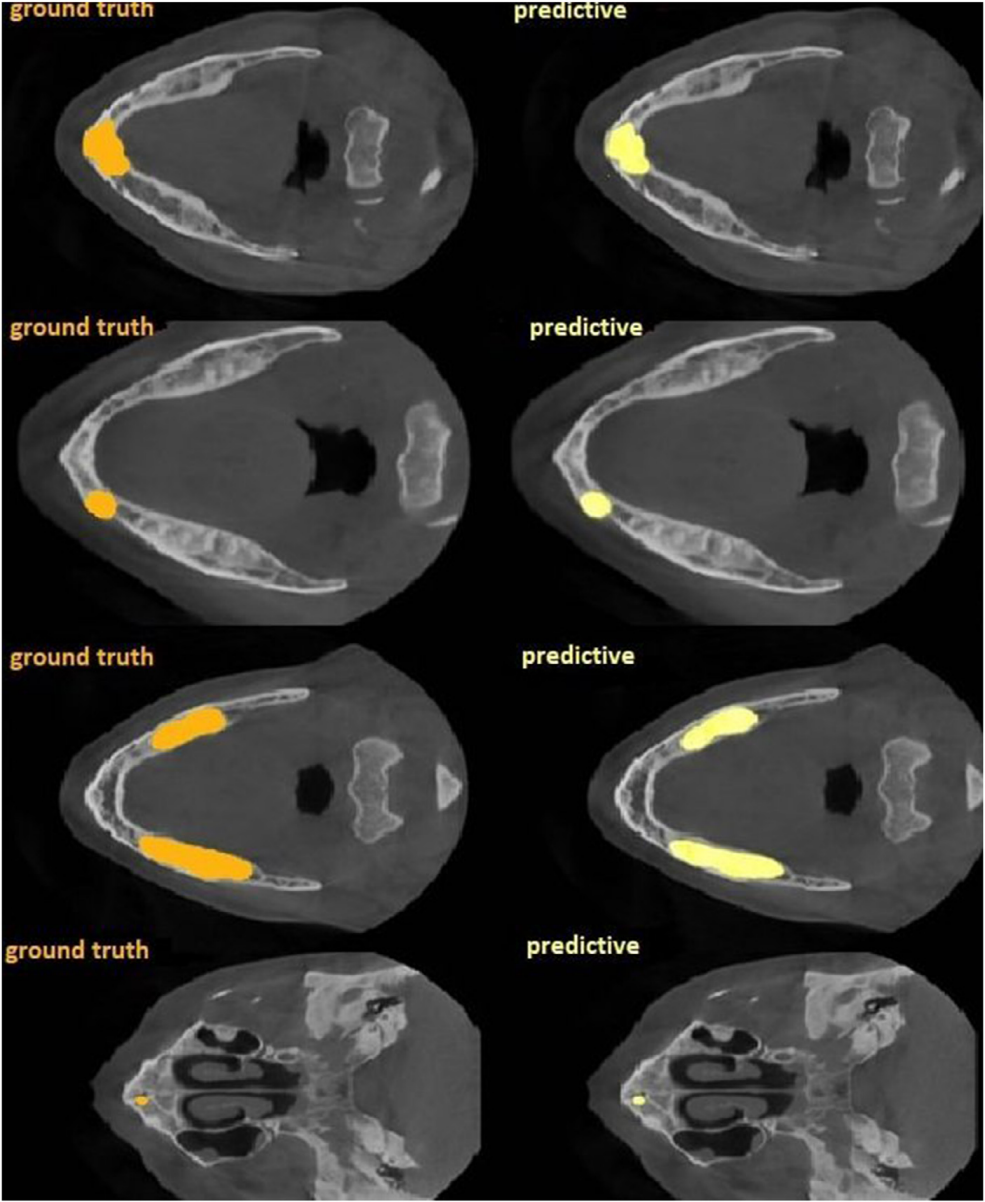
Ground truth and predicted segmentation images on CBCT axial slices.

**Fig. 7:**
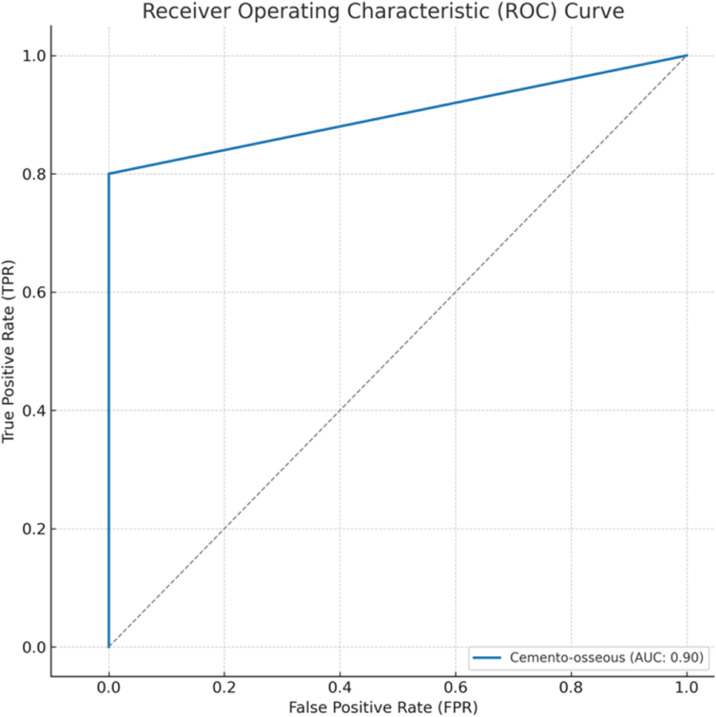
ROC curve and AUC of the nnU-Net v2 model for cemento-osseous dysplasia segmentatio.

**Fig. 8:**
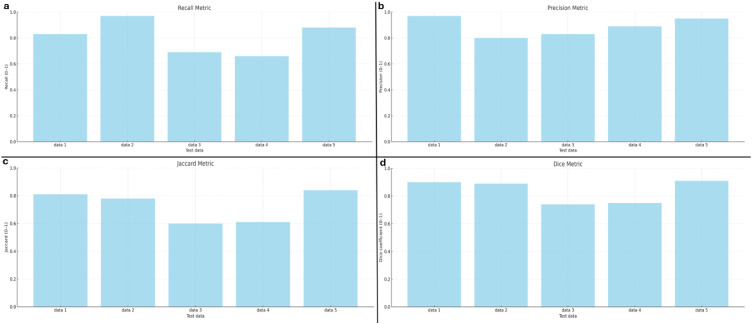
Graphs of the recall A, precision B, jaccard C, and dice metrics D, derived from the test data.

As a result of cemento-osseous dysplasia segmentation, the nnU-Net v2-based algorithm successfully predicted voxels belonging to cemento-osseous dysplasia with more than 50% intersection in almost all samples (Figure 6). The sensitivity, precision, Dice coefficient and Jaccard index values were 0.805, 0.889, 0.839, and 0.730, respectively (Table 1). The AUC of the nnU-Net v2 model for cemento-osseous dysplasia segmentation was 0.90 (Figure 7). The precision, recall, Dice coefficient and Jaccard index graphs obtained from the test results of the cemento-osseous dysplasias are shown in Figure 8.

**Table 1:** Performance measurement values obtained from test data of nnU-Net V2.

	Sensitivity	Precision	Dice coefficient	Jaccard indeks
Cemento-osseous dysplasia segmentation	0.805	0.889	0.839	0.730

## Discussion

Although “cemento-osseous dysplasia” is a histopathologic term, the diagnostic process is usually based on a holistic evaluation of demographic, clinical, radiologic and follow-up data.^11^ Invasive dental procedures such as biopsies are generally contraindicated, as cement-like deposition and reduced local vascularization render these lesions susceptible to infections.^11^ For this reason, it is of great importance to know the exact radiologic signs of these lesions. Nemec et al.^2^ emphasized that the follow-up of cemento-osseous dysplasias is a difficult issue to address. Currently, there are no guidelines on how long and how often patients should be followed given the long-term nature of cemento-osseous dysplasias.^2^ This situation has resulted in lesion follow-up being at the discretion of clinicians. Tracking can be standardized and objectively evaluated using systems integrated with AI algorithms. As highlighted by Alahmari et al.,^18^ segmentation-based digital image analysis constitutes a fundamental step in the development of pathology-oriented AI diagnostics, aligning closely with the objectives of the present study, which focuses on the radiologic evaluation of cemento-osseous dysplasias.

The diagnostic capabilities of deep learning algorithms for identifying of radiopaque or mixed jaw lesions have been investigated in the literature have been investigated. There is a significant gap in the literature regarding the diagnostic potential of these technologies for radiopaque or mixed lesions, including other lesions such as odontoma, osteoma, or osteomyelitis.^19^ In this study, cemento-osseous dysplasias, for which radiological diagnosis and follow-up are of great importance, were segmented with an AI model.

It has been reported that cemento-osseous dysplasias can sometimes be confused with other single or multiple radiopaque lesions described as idiopathic osteosclerosis, dense bone islands or enostosis.^12^ These lesions, of unknown etiology, are well-defined, typically round or oval, and sometimes irregularly shaped. They are distinguished from the mature stage of cemento-osseous dysplasias by the absence of a thin radiolucent margin. Taşsöker et al.^19^ used a YOLOv5-based AI model for the detection of idiopathic ostosclerosis on panoramic radiographs. With 0.981 precision and 0.929 recall values, the developed model showed a successful performance. In another study which idiopathic osteosclerosis was detected with an AI-based model, GoogLeNet Inception v2 architecture was used on 493 panoramic radiographs.^20^ It has been reported that the algorithm used has the potential to accurately detect idiopathic osteosclerosis with a sensitivity of 0.88 and an accuracy of 0.83.

In this study, cemento-osseous dysplasias were segmented on CBCT images and successful results were obtained with a precision of 0.889 and a sensitivity of 0.809. Although the number of data used in this study was small, the model's diagnostic performance was successful probably for 2 reasons. The segmentation being performed on CBCT images without superposition and in 3 dimensions, and the ability of the nnU-Net architecture was used to facilitate automatic configuration for various datasets with minimal experimental selection requirements through a simple set of operations. This architecture is characterized by fast execution of tasks and requires minimal computational resources beyond the initial model training phase. Consequently, nnU-Net exhibited high data efficiency. It has shown efficient results in various partitions from large datasets as well as in cases with limited training data.^21^

Depending on the stage of cemento-osseous dysplasias, the differential diagnosis can range from cystic or inflammatory periodontal lesions to some odontogenic tumors. In the literature, there are studies in which lesions seen in the jaw bones that can be confused with the radiolucent appearance of cemento-osseous dysplasias, especially in the osteolytic stage, were diagnosed with AI algorithms on periapical, panoramic and CBCT images.^22^, ^23^, ^24^, ^25^ In this study, images of cemento-osseous dysplasias in the osteolytic stage, which exhibit a radiolucent appearance and may be confused with periapical lesions, were not used. Clinical examination is also required to differentiate this radiolucent appearance from periapical lesions. This study was designed to test whether the deep learning model can be used as an automated radiological prediagnostic decision support mechanism, in which, images characterized by the specific appearance of the mixed stage of cemento-osseous dysplasias (mixed images containing radiopaque foci surrounded by a radiolucent halo), which oral radiologists can diagnose cemento-osseous dysplasias radiologically, were used. In line with the observations of Tuygunov et al.,^26^ the integration of AI-based tools into radiological workflows may improve diagnostic efficiency and support clinicians in complex image interpretation tasks.

After segmentation, the Dice coefficient and Jaccard index were 0.839 and 0.730, respectively. The Jaccard index was used to measure the similarity between the ground truth and the estimated segmentation area. In the test results, when the lesions where the labeled ground truth area and the segmentation area do not match are examined, it can be said that the lesions that merge with the roots of the teeth and the lesions cannot be fully separated from the teeth during labeling, which reduces the performance of the model. Segmentation of the teeth prior to lesion detection can improve the performance of AI algorithms in diagnosing cemento-osseous and other lesions that surround the tooth roots.

In the literature, there are also studies in which cysts and tumors were detected and classified using AI algorithms with panoramic and CBCT images other than periapical lesions.^7^^,^^27^, ^28^, ^29^ In a review of studies using machine learning to diagnose maxillofacial cysts and tumors in the literature, it was reported that the average sensitivity and specificity of studies using panoramic radiographs were 0.83 and 0.82, respectively, while studies using CBCT showed superior performance with sensitivity and specificity values of 0.88 and 0.88, respectively.^30^ Cemento-osseous dysplasias are usually asymptomatic, but some cases with accompanying symptoms have also been observed.^31^ Cases have been reported in which simple and aneurysmal bone cysts and nonspecific cystic degenerations have been associated with florid cemento-osseous dysplasia and less frequently with periapical cemento-osseous displasia types.^32^^,^^33^ However, the infection that develops within the cemento-osseous dysplasia, namely osteomyelitis, is the most significant complication that can be encountered.^31^ Radiologic features of osteomyelitis can be observed similar to cemento-osseous lesions, especially floride cemento-osseous dysplasia.^34^ In the literature, there are also studies in which automatic detection of osteomyelitis with jaw cysts and tumors is performed. Using the WaveletFusion-ViT model as a semi-supervised learning method for the classification of chronic suppurative osteomyelitis with ameloblastoma and periapical cysts, Liang et al.^35^ used panoramic radiographs generated from 385 CBCT images.

Liu et al.^36^ proposed an automatic deep learning method based on a modified nnU-Net model for classification and 3-dimensional segmentation of 5 jaw lesions including ameloblastoma, odontogenic keratocyst, dentigerous cyst, periapical cyst and osteomyelitis. They also reported that it can help physicians improve classification accuracy and segmentation efficiency in differentiating some lesions. The nnU-Net v2-based model used in this study successfully segmented cemento-osseous dysplasias, but the performance of the intelligence model was not compared with the diagnostic performance of radiologists.

This study hads some limitations. The images used were obtained from a single center and represent a relatively narrow patient population. This may limit the generalizability of the findings, and obtaining training and test sets from the same device may pose a risk of overfitting. Only images from mixed-stage cemento-osseous dysplasia were included, and lesions were segmented collectively rather than subtyped. Furthermore, this study did not assess interobserver variability and direct comparisons between AI and human experts. This limitation is explicitly acknowledged, and future research should include multiexpert assessments to better compare human and AI segmentation performance. Furthermore, cross-validation was not implemented in this study; therefore, performance metrics may vary depending on the sample distribution. Future studies with larger, multicenter datasets could incorporate k-fold cross-validation methods to improve the reliability and generalizability of the model.

## Conclusion

This study yielded promising results for the segmentation of cemento-osseous lesions in CBCT images using an AI model based on nnU-Net v2. These findings suggest that this approach could improve the detection and monitoring of cemento-osseous dysplasias, where radiologic evaluation is essential for clinical decision-making.

## Informed consent

The need for informed consent was waived because of the retrospective nature of this study.

## Ethical approval

All procedures performed in studies involving human participants were in accordance with the ethical standards of the institutional and/or national research committee and with the 1964 Helsinki Declaration and its later amendments or comparable ethical standards. The study was approved by the Inonu University Non-Interventional Clinical Research Ethics Committee decision dated 31.10.2023 and numbered 2023-5087. Before each CBCT scan, written informed consent was obtained from all participants included in the study.

## Declaration of generative AI and AI-assisted technologies in the writing process

During the preparation of this work, the authors used ChatGPT (OpenAI) to assist with language editing and improving clarity. After using this tool, the author(s) reviewed and edited the content as needed and take full responsibility for the content of the published article.

## Author Contributions

**Duygu Çelik Özen:** Writing – review & editing, Writing – original draft, Supervision, Resources, Project administration, Methodology, Investigation, Funding acquisition, Data curation, Conceptualization. **Oğuzhan Altun:** Writing – review & editing, Writing – original draft, Methodology, Formal analysis, Data curation. **Şuayip Burak Duman:** Writing – review & editing, Methodology, Investigation. **İbrahim Şevki Bayrakdar:** Writing – review & editing, Methodology, Investigation.

## Funding

This study was supported by Scientific Research Projects Coordination Unit of Inonu University (project # TDH-2024-3370), Malatya, Turkey.

## Data availability

The data that support the findings of this study are available from the corresponding author upon reasonable request.

## Declaration of competing interest

The author is an Editorial Board Member/Editor-in-Chief/Associate Editor/Guest Editor for this journal and was not involved in the editorial review or the decision to publish this article.
